# CD4^+^ and CD8^+^ T cells contribute to psoriasis phenotype when integrated into human skin equivalents for drug testing

**DOI:** 10.3389/fimmu.2026.1790531

**Published:** 2026-04-10

**Authors:** Irit Vahav, Maria Thon, Jasper J. Koning, Susan Gibbs, Andreas Thiel, Lucie Loyal

**Affiliations:** 1 Berlin Institute of Health (BIH) at Charité – Universitätsmedizin Berlin, Center of Immunomics - Regenerative Immunology and Aging, Berlin, Germany; 2 Department of Molecular Cell Biology and Immunology, Amsterdam UMC Location Vrije Universiteit Amsterdam, Amsterdam, Netherlands; 3 Amsterdam Movement Sciences, Tissue Function & Regeneration, Amsterdam UMC Location Vrije Universiteit Amsterdam, Amsterdam, Netherlands; 4 Amsterdam Institute for Immunology and Infectious Diseases, Amsterdam UMC Location Vrije Universiteit Amsterdam, Amsterdam, Netherlands; 5 Department of Oral Cell Biology, Academic Centre for Dentistry Amsterdam (ACTA), University of Amsterdam and Vrije Universiteit, Amsterdam, Netherlands; 6 Si-M/”Der Simulierte Mensch”, a science framework of Technische Universität Berlin and Charité – Universitätsmedizin Berlin, Berlin, Germany; 7 Charité - Universitätsmedizin Berlin, Corporate Member of Freie Universität Berlin and Humboldt-Universität zu Berlin, Institute of Medical Immunology, Berlin, Germany

**Keywords:** CD8+ T cells, human skin equivalent, inflammation, keratinocytes, psoriasis

## Abstract

The limited translational potential of experimental animal models for human skin disease pathophysiology demands the development of more suitable immunocompetent human experimental models. Psoriasis ranks among the most prevalent human inflammatory skin disorders, initiated and manifested by the interplay of T cells with epidermal keratinocytes. Current *in vitro* models recapitulate psoriasis-like pathology by incorporating CD4^+^ T cell subsets into skin equivalents, but lack CD8^+^ T cells, key mediators of epidermal inflammation and chronic disease. Our aim was to establish a human experimental model incorporating different CD4^+^ and CD8^+^ T cell subsets into a human full-thickness skin equivalent (hFTSE) to investigate their individual and combined contribution to the pathogenesis of psoriasis. Across all conditions, migration of inserted T cells towards the epidermis and the secretion of psoriasis-associated cytokines (IFN-γ, IL-17, TNF-α, IL-1β, CCL20, IL-6, and IL-10) was observed. Immunohistological analysis revealed psoriasis-associated alteration of epidermal differentiation, as well as enhanced expression of keratinocyte-mediated antimicrobial peptides (AMPs) in T cell-incorporated hFTSEs. Notably, the presence of CD8^+^ T cells altered the efficacy of treatment with the psoriasis drug apremilast. Overall, our findings contribute to the refinement of human skin models for studying disease pathophysiology and evaluating therapeutic responses, enabling more accurate predictions of drug efficacy and safety.

## Introduction

Psoriasis is a chronic autoinflammatory skin disorder characterized by erythematous, dry and scaly plaques and is frequently associated with systemic comorbidities including psoriatic arthritis, type 2 diabetes and cardiovascular diseases, affecting approximately 1-3% of the Western population (1). Histopathological features of psoriatic lesions include epidermal hyperplasia, dermal vasodilation, immune cell infiltrations and disrupted skin barrier integrity. Psoriasis is associated with a genetic predisposition in combination with exposition to distinct toxins, microbiome dysbiosis or skin injuries (2). A central pathomechanism in psoriasis is an altered cytokine-mediated crosstalk between epidermal keratinocytes and different T cell subsets in the skin. Damage-associated molecular patterns (DAMPs) or complexes formed by AMPs and self-DNA released by damaged keratinocytes may activate plasmacytoid dendritic cells (pDCs), which, in turn, can promote the activation of autoantigen-specific T cells secreting IFN-γ, TNF-α, IL-17 and IL-22 (3–5). In particular, T cell-derived TNF-α and IL-17 can then induce hyperproliferation and altered keratinocyte differentiation, as well as modify the composition of skin-barrier proteins (6, 7). In turn, activated keratinocytes secrete inflammatory mediators and chemoattractants such as CCL20, which further attract and promote T cell differentiation and infiltration into psoriatic lesions, thereby resulting in recurrent inflammation and chronic disease (8).

The plasticity of CD4^+^ T cells to differentiate into T-helper (Th) subsets - such as type 1 (Th1) or type 17 (Th17) cells, which produce the key inflammatory cytokines IFN-γ and IL-17, respectively - is well established, and their role in psoriasis has been thoroughly investigated (3, 5, 9). However, the identification of psoriasis-related autoantigens (LL-37, ADAMTSL5) presented on MHC class I allele HLA-Cw6 - a major genetic risk factor - emphasized a critical role for CD8^+^ T cells in the initiation and progression of psoriatic inflammation (10–12). Accordingly, depletion of CD8^+^ T cells prevents psoriasis development in a xenotransplantation mouse model (13). In the last decades it has been shown that the differentiation capacity of CD8^+^ cytotoxic T cells (Tc) extends beyond the classical IFN-γ-producing Tc1 cells and includes IL-17-secreting Tc17 CD8^+^ T cells (13–17). Tc17 cells are enriched in peripheral blood of psoriasis patients, and CD8^+^ T cells represent the dominant T cell population in both acute and resolved psoriatic lesions (16, 18). Moreover, we have shown that human Tc17 cells lack classical cytotoxic features and instead expresses the CD4^+^ helper molecule CD40L (19). These findings emphasize the need for relevant human *in vitro* models to elucidate the pathophysiological roles of CD8^+^ T cells - including the Tc17 subset - in the pathogenesis of diseases such as psoriasis.

Most approved psoriasis therapeutics target cytokine or T cell-mediated immune activation, including broad immunosuppressants (e.g. methotrexate), small molecules (e.g. apremilast) or more specific biologicals (e.g. anti-IL-17/anti-TNF-α/anti-IL-23 antibodies) (20–22). However, the high relapse rates after treatment discontinuation underline a limitation of current treatment strategies (23). A challenge is the lack of a detailed understanding of the distinct contributions of CD4^+^ and CD8^+^ T cells to disease initiation, localization and progression in most *in vitro* studies to date (24, 25). Moreover, while numerous murine models of psoriasis exist, their translational relevance is limited due to differences in immune cell composition and morphological properties of murine and human skin and skin:microbiome interactions in specific pathogen free (SPF) mice (26, 27). To overcome these limitations, three-dimensional (3D) human skin models have been developed to mimic native skin morphology and enable the study of individual pathological cell functionalities and cell–cell interactions in psoriatic inflammation (28). Current state of the art human full-thickness skin equivalents (hFTSEs), consisting of a dermal layer overlaid by a differentiated and stratified epidermis, with incorporated psoriasis-associated CD4^+^ T cells, have been shown to recapitulate the infiltration of inflammatory T cells towards chemoattractant-producing epidermal keratinocytes and the cytokine mediated induction of psoriasis (29, 30). Here, we extend the complexity of existing 3D skin models by integrating psoriasis-related CD8^+^ T cell subsets to investigate the interplay between different skin cells and distinct T cell subpopulations in psoriasis pathogenesis. By independently or jointly incorporating CD4^+^ (Th1, Th17) and CD8^+^ (Tc1, Tc17) T cell subsets, we delineate distinct and synergistic contributions to psoriasis induction. Both T cell subpopulations can infiltrate the hFTSE and upregulate hallmark psoriatic markers; however, responsiveness to the psoriasis drug apremilast differs between the T cell subsets. Our advanced, immunocompetent skin model provides a valuable platform for studying T cell migration and residency and for evaluating the safety and efficacy of therapeutic agents in a physiologically relevant setting.

## Materials and methods

### Human tissue and blood

Whole blood from healthy donors was collected in lithium heparin tubes and peripheral blood mononuclear cells (PBMCs) were isolated by gradient density centrifugation according to the manufacturer’s instructions (Biocoll, Bio&Sell, Nürnberg, Germany). Human keratinocytes and dermal fibroblasts were isolated from juvenile foreskin as previously described (31). Human juvenile foreskin was obtained from healthy individuals undergoing circumcision. Human blood was obtained from healthy volunteers. All skin and blood samples were obtained in accordance with the relevant regulations, with informed consent and ethical approval from the from the Ethics Committee of the Charité Universitätsmedizin, Berlin, Germany (EA2/240/23 and EA2/091/12, 29) or Ärztekammer Berlin, Germany (Eth-10/15).

### Generation of full thickness human skin equivalents

Cell culture of epidermal keratinocytes and dermal fibroblasts as well as the construction of hFTSE was carried out as previously described (32) and cultured at 37 °C, 5% CO_2_. Fibroblasts were cultured in DMEM (Corning, Corning, NY, USA) with 5% Fetal Clone III (HyClone, Logan, UT, USA) and 1% penicillin/streptomycin (Gibco, Waltham, MA, USA). Epidermal keratinocytes were amplified in DermaLife medium (Lifeline Cell Technology, Frederick, MD, USA) with 1% penicillin/streptomycin and adapted to keratinocyte medium-I (DMEM/Ham’s F12 in a 3:1 ratio supplemented with 5% Fetal Clone III, 1 μmol/L hydrocortisone (Sigma-Aldrich, St. Louis, MO, USA), 1 μmol/L isoproterenol hydrochloride (Sigma-Aldrich), 0.1 μmol/L insulin (Sigma-Aldrich), 2 ng/mL human keratinocyte growth factor (Sigma-Aldrich), and 1% penicillin/streptomycin). hFTSEs were constructed in a Millicell^®^ insert of 9 mm inner diameter and 0.4 μm pore size (Merck, Darmstadt, Germany). 300 µL of hydrogel mixture, comprised of 3 mg/ml collagen (Ibidi, Gräfelfing, Germany), 1 mg/ml fibrinogen (Enzyme Research Laboratories, Inc., South Bend, IN) and 0.5 U/ml thrombin (Merck) was populated with fibroblasts (6.5*10^4^ cells/ml) and cultured for one day submerged in keratinocyte medium‐I without human keratinocyte growth factor. On the following day 8*10^4^ keratinocytes were seeded on top of the fibroblast populated hydrogels and further cultured submerged in keratinocyte medium-I. After four days of submerge culture the epidermal side of the hFTSE was exposed to the air and cultured at air liquid interface for 9–10 days in keratinocyte medium-II (DMEM/Ham’s F12 in a 3:1 ratio containing 1% Fetal Clone III, 1 μmol/L hydrocortisone, 1 μmol/L isoproterenol hydrochloride, 0.1 μmol/L insulin, 10 μmol/L L-carnitine (Sigma-Aldrich), 10 mmol/L L-serine (Sigma-Aldrich), 50 μg/mL ascorbic acid (Sigma-Aldrich), and 1% penicillin/streptomycin.

### *In vitro* polarization of naïve T cells

The protocol for T cell polarization was partially adopted from a previous study (30) and modified as follows; 24-well plates (Thermo Fisher Scientific, Waltham, MA, USA) were coated with 480 µL PBS (Gibco) containing 1 µg/mL anti-CD3 (clone UCHT1, BD Pharmingen, San Diego, CA, USA) and 3 µg/mL anti-CD28 (clone CD28.2, BD Pharmingen) for Type1 polarization, or 1 µg/mL anti-CD3 for Type17 polarization and incubated for 2 hours at 37 °C prior to cell seeding. Naïve CD4^+^ and CD8^+^ T cells were purified from PBMCs using magnetically enriched naïve T cells isolation kits according to the manufacturer’s instructions (Miltenyi Biotec, Bergisch Gladbach, Germany). Freshly isolated naïve CD4^+^ and CD8^+^ T cells were seeded into 24-well plates and cultivated in T cell medium (TCM) containing (RPMI medium 1640 (Gibco) with 10% Fetal Clone III and 1% penicillin/streptomycin). Type 1 polarization was conducted with medium containing 20 ng/ml IL-12, 5 µg/ml anti–IL-4 (both Miltenyi Biotec) and 2 mL of 2*10^6^ cells/mL naïve CD4^+^ T cells or 1 mL of 0.7*10^6^ cells/mL naïve CD8^+^ T cells were seeded. Type 17 polarization was conducted with medium containing 20 ng/ml IL-1β, 40 ng/ml IL-6, 5 µg/ml anti–IL-4 (all Miltenyi Biotec), 10 µg/ml anti–IFN-γ (clone B27, BD Pharmingen), and 5 µg/ml anti-CD28 and 1 mL of 0.33*10^6^ cells/mL naïve CD4^+^ T cells or 1 mL of 0.7*10^6^ cells/mL naïve CD8^+^ T cells were seeded. Non-polarized controls were cultivated at 1 mL of 5-7*10^5^ cells/mL with 10 ng/ml IL-7 and 10 ng/ml IL-15 (both Miltenyi Biotec). After 48 hours of incubation at 37 °C, 5% CO_2_ all cell populations were transferred into an uncoated 24-well plate and supplemented with 300 U/ml IL-2 (Miltenyi Biotec) for Th1 and Tc1 cultures and 100 U/ml IL-2 and 40 ng/ml IL-23 (Miltenyi Biotec) for Th17 and Tc17 cultures. Cell cultures were further incubated until day 6 and then restimulated for 6 hours with 10 ng/ml phorbol-12-myristat-13-acetat (PMA) and 1 µg/mL ionomycin (both Sigma-Aldrich) before T cell phenotype analysis. All restimulations were carried out in TCM at a cell concentration of 1*10^6^ cells/mL.

### T cell integration into human skin equivalents

Polarized Th1, Th17, Tc1 and Tc17 subsets were restimulated for 6 hours in plates precoated with 1 µg/mL anti-CD3 (BD Pharmingen) and TCM containing 5 µg/mL anti-CD28 (BD Pharmingen) prior to their integration into hFTSEs. T cells were seeded into Millicell^®^ inserts (9 mm inner diameter and 0.4 μm pore size) with a total of 1.5*10^5^ cells/insert. To generate three types of T cell-bearing hFTSE constructs, T cell subsets were seeded in the following combinations: 7.5*10^4^ cells each of Th1 and Th17 (hFTSE+Th1/17); 7.5*10^4^ cells each of Tc1 and Tc17 (hFTSE+Tc1/17); 5*10^4^ cells each of Th1 and Th17 and 2.5*10^4^ cells of each Tc1 and Tc17 (hFTSE+Tc1/17+Tc1/17). Next, 9–10 days air-exposed hFTSEs were transferred from their inserts directly onto the T cell layers and co-cultured for 3 days. T cell-free control cultures, representing non-psoriatic hFTSE, were handled identically to T cell-bearing hFTSEs and transferred onto T cell-free inserts to assess baseline inflammation potentially induced by the transfer procedure. Additional T cell-free hFTSE constructs were maintained in their original inserts as non-transfer control to assess the level of inflammatory cytokine release without insert replacement. For comparative analyses between hFTSEs cultured in the presence or absence of T cells, only T cell-free control cultures were used as the reference condition. All hFTSE constructs were placed in a 6-well plate and cultured with 0.9 mL 1:1 Keratinocyte medium-II: TCM and further cultured under air-exposed condition for 3 days of co-culture at 37 °C, 5% CO_2_.

### Anti-psoriasis drug testing on human skin equivalents

At the time of T cell integration, triplicates of each hFTSE condition, including T cell-free control hFTSE cultures, were treated with an anti-psoriasis drug, while parallel triplicates of the same condition were left untreated. Apremilast (AdooQ Bioscience, Irvine, CA, USA) was administered at 2.5 µM directly into the culture medium. After 48 hours of co-culture, 100 µL of culture medium was replaced in all cultures; drug-treated conditions were replenished with medium supplemented with 2.5 µM apremilast. On day 3 of co-culture, all constructs were harvested for histological analysis, and supernatants were collected and stored at –20 °C until further analysis.

### Flow cytometry

To assess the purity of the enriched naïve CD4^+^ and CD8^+^ T cells populations, T cells were stained with the following fluorochrome-conjugated antibodies titrated to their optimal concentration: CD3-Alexa700 (clone UCHT1, BioLegend, San Diego, CA, USA), CD8-PerCP (clone SK1, BioLegend), CD4-BrilliantViolet605 (clone RPA-T4, BioLegend), CCR7-AlexaFluor488 (clone G043H7, BioLegend), CD45RA-APC-H7 (clone HI100, BD Pharmingen). Samples were measured on a MACSQuant 16 Analyzer (Miltenyi Biotec) and analyzed with FlowJo v10 (BD Pharmingen).

### Quantitative PCR

*In vitro* polarized T cells were collected, lysed in RA1 lysis buffer (Macherey-Nagel, Düren, Germany) and stored at −80 °C. mRNA isolation was carried out using the NucleoSpin RNA Kit (Macherey-Nagel) according to the manufacturer’s instructions. cDNA was generated utilizing TaqMan Reverse Transcription kit according to the manufacturers protocol (Applied Biosystems by Thermo Fisher Scientific,Waltham, MA, USA). Real‐time polymerase chain reaction was performed using the LightCycler^®^ 480 system (Roche, Basel, Switzerland) with Applied Biosystems™ TaqMan™ Fast Advanced Master Mix and predesigned TaqMan™ (FAM-labeled) Gene Expression Assays (Thermo Fisher Scientific), according to the manufacturer’s instructions. The following TaqMan™ probes were used: IFN-γ (Hs00989291_m1), IL-17A (Hs00174383_m1), IL-17F (Hs00174383_m1), IL-4 (Hs00174122_m1), IL-13 (Hs00174379_m1), IL-22 (Hs01574154_m1) and TBP (Hs00427620_m1). Fold change expression in the target gene relative to the TBP housekeeping gene was calculated with the LightCycler^®^ 480 software (version 1.5.1.62 SP3), using the advanced relative quantification analysis and 2^nd^ Derivative Maximum method.

### Cytokine secretion analysis

Supernatant of T cell and hFTSE cultures were collected at the time of end point analysis and stored at −20 °C until further analysis. Cytokine concentration in cell culture supernatant was analyzed using enzyme‐linked immunosorbent assay (ELISA) for IFN-γ, IL-17A, IL-1β, and IL‐6 (Mabtech, Nacka Strand, Sweden) and CCL20 and TNF-α (R&D Systems, Minneapolis, MN, USA) in accordance with the manufacturer’s specifications. Absorbance was measured at 450 nm using Infinite^®^ M Plexplate reader (Tecan, Männedorf, Switzerland) and data analyzed using the Magellan Pro (version 7.4, Tecan) data analysis software. IL-10 was measured using LEGENDplex™ bead-based immunoassay, Human Inflammation Panel 1 (BioLegend). Legendplex immunoassays were performed in accordance with the manufacturer’s specifications, measured on an Attune Nxt (Thermo Fisher Scientific) and analyzed using LEGENDplex™ Data Analysis Software Suite (version 2024-06–15 BioLegend). If the concentration of all intra-triplicate analytes was undetectable in the ELISA analysis, the detection limits (as indicated in the manufacturer’s specifications) were used to calculate comparison to control.

### Histology, immunohistochemistry, and immunofluorescent staining

hFTSEs were fixed in 4% formaldehyde overnight at room temperature and embedded in paraffin. Paraffin embedded tissue sections (5 μm) were used for morphological (hematoxylin and eosin staining, H&E), immunohistochemical and immunofluorescent characterization as previously described (32, 33). Immunohistochemistry staining was performed using cytokeratin-10 (clone DE-K10, Progen, Heidelberg, Germany), loricrin (clone Poly19051, BioLegend), Beta-defensin 2 (clone L12-4C-C2, Origene, Rockville, MD, USA), Elafin/SKALP (clone TRAB20, hycultBiotech Uden, The Netherlands). Immunofluorescent staining was performed using CD3 (FLEX RTU, Agilent, Santa Clara, CA) and fluorophore-conjugated secondary antibody CF^®^488A Goat anti-Rabbit (Biotium, Fremont, CA) and DAPI (Abcam, Cambridge, UK). Zeiss AxioObserver Z1 and Zeiss AxioScope 5 microscopes (Zeiss, Oberkochen, Germany) were used for immunofluorescence-, H&E as well as immunohistochemical- imaging, and analyzed with Zen software (blue edition, version 3.4.91 Zeiss).

### Statistical analysis

Four independent experiments were performed, each representing different donors of T cells and skin cells (donor-matched fibroblast and keratinocytes) with an intra-experiment triplicate. These triplicate values were averaged within an experiment to obtain 1 data point per experiment. Data are presented as mean values and standard error of the mean (± SEM) of the 4 experiments using GraphPad Prism 10 software (version 10.3 GraphPad Software Inc., La Jolla, CA, USA).

## Results

### *In vitro* polarization of psoriasis-associated T cell subsets

**Figure 1:**
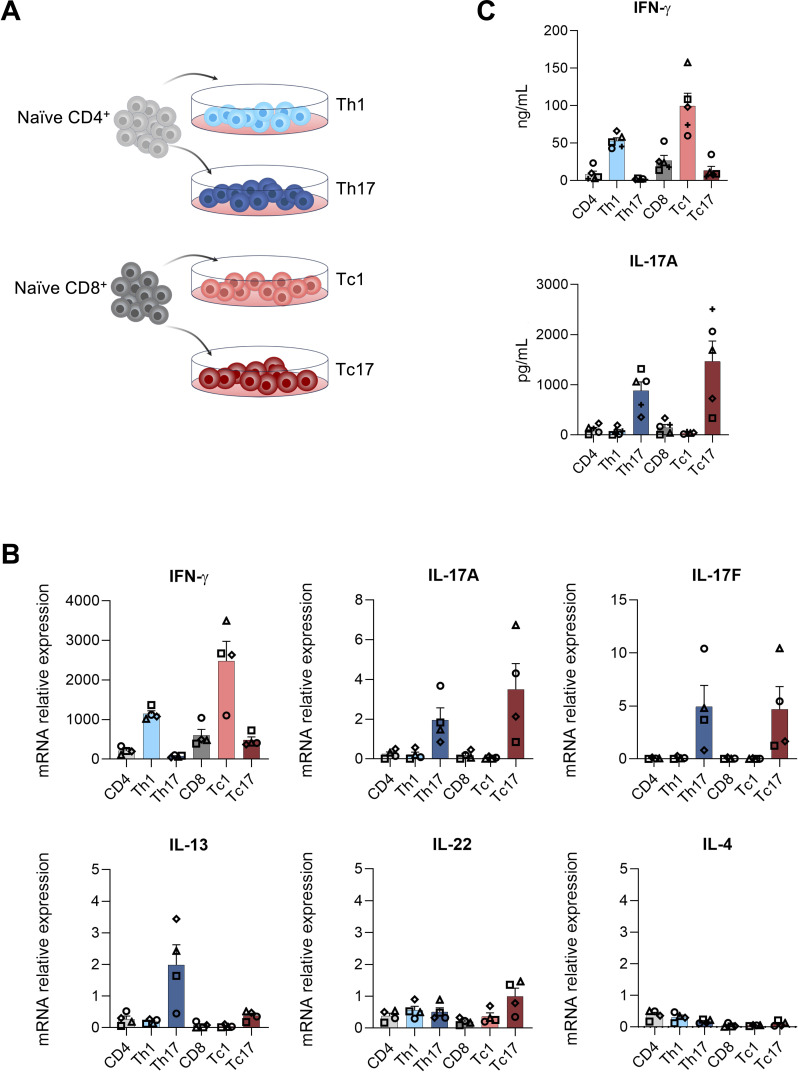
Psoriasis-like cytokines expression profile of *in vitro* polarized CD4^+^ and CD8^+^ T cell subsets. **(A)** Schematic representation of *in vitro* polarization of naïve CD4^+^ and CD8^+^ T cells. Naïve T cells were differentiated into Th1, Th17, Tc1 and Tc17 subsets by cultivation in cytokine cocktail media for 6 days and restimulated with PMA/ionomycin prior to analysis. **(B)** qRT-PCR based quantification of psoriasis-associated cytokine IFN-γ, IL-17A, IL-17F, IL-13, IL-22, and IL-4 in Th1, Th17, Tc1 and Tc17 cultures compared to control cultures (n=4). **(C)** Titers of IFN-γ and IL-17A cytokines in the supernatant of the T cell cultures (n=5). Bar graphs show mean ± SEM values. Bar-colors represent individual T cell subsets (light grey naïve CD4^+^, light blue Th1, dark blue Th17, dark grey naïve CD8^+^, light red Tc1, dark red Tc17).

Naïve CD4^+^ and CD8^+^ T cells were differentiated *in vitro* into type 1 and type 17 T cells to analyze their role in inducing psoriasis-like inflammation in hFTSEs (**Figure 1A**). Polarization efficiency was assessed using naïve CD4^+^ and CD8^+^ T cells cultivated under homeostatic conditions (IL-7/IL-15). After 6 days of polarization, Th1- and Tc1-subsets expressed high levels of IFN-γ, the central type 1 T cell cytokine, while the Th17 and Tc17 subsets showed increased levels of the type 17 cytokines, IL-17A and IL-17F (**Figures 1B, C**). IL-13 was elevated in Th17 and Tc17 subsets, while IL-22 was slightly increased in the Tc17 subset. None of the conditions induced the expression of the central type 2 cytokine IL-4 (**Figure 1B**). In line with the mRNA expression levels, ELISA analysis of cytokine release in T cell culture supernatants showed increased IFN-γ and IL-17A release in Th1, Tc1 and Th17, Tc17 cultures, respectively (**Figure 1C**).

### Combined integration of psoriasis-associated CD4^+^ and CD8^+^ T cell subsets into human skin equivalents

**Figure 2:**
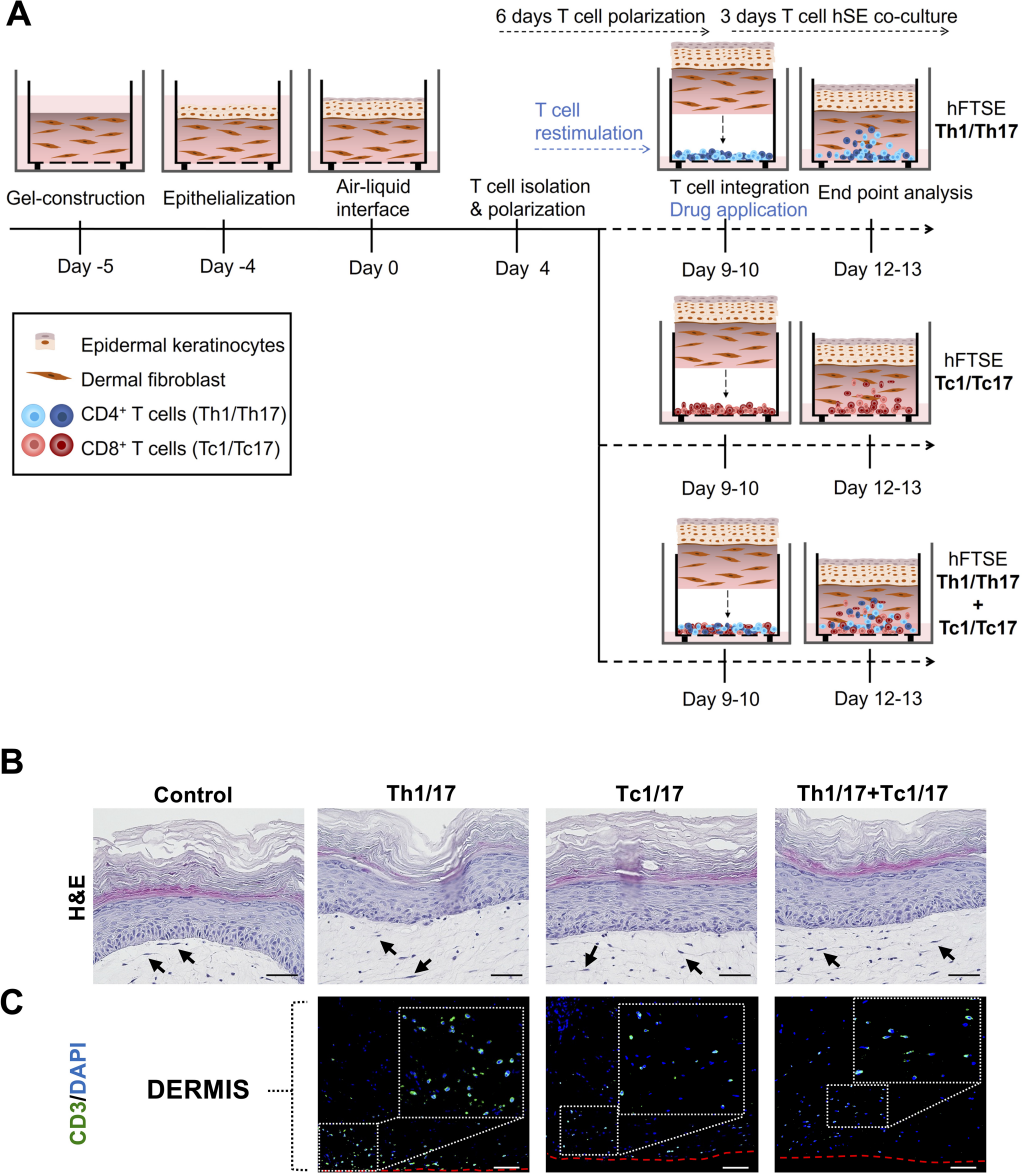
Integration of psoriasis-associated T cells into human full thickness skin equivalents. **(A)** Experimental design for hFTSE containing psoriasis related T cell subsets. Following fibroblast-populated hydrogel construction and epithelialization the human skin equivalents were air-exposed for 9–10 days prior to incorporation of activated T cells in three different combinations: CD4^+^ T cell subsets (Th1/17), CD8^+^ T cell subsets (Tc1/17) or combined CD4^+^ and CD8^+^ T cells (Th1/17+Tc1/17). After 3 days of co-culture the hFTSEswere analyzed. **(B)** Morphological characterization of hFTSEs with or without polarized T cells by Hematoxylin–eosin staining. The black arrows indicate elongated dermal fibroblasts in hydrogel matrix. Scale bar = 50 µm. **(C)** CD3^+^ (green) expressing Th1/17 and Tc1/17 cells are observed in the dermis migrating towards the epidermis of the hFTSEs. The red dashed line indicates hydrogel bottom. DAPI (blue) stains cell nuclei. Scale bar = 100 μm.

Both CD4^+^ and CD8^+^ T-lymphocytes are key players in the pathogenesis of psoriasis. Therefore, we generated three distinct hFTSE constructs integrated Th1/7 CD4^+^ T cells and Tc1/17 CD8^+^ T cells separately and in combination into hFTSEs to assess their potential to induce psoriatic inflammation. hFTSEs composed of fully differentiated and stratified epidermis on a fibroblast populated hydrogel were co-cultured for 3 days with the T cell subsets (**Figure 2A**). Following T cell integration, hFTSE constructs maintained structural tissue integrity, characterized by intact dermal matrix populated by spindle-shaped fibroblasts (black arrows), overlaid by a differentiated epidermis with a cornified layer (**Figure 2B**). The CD3^+^ (in green) expressing Th1/17 and Tc1/17 T cell populations migrated from the insert:hFTSE intersection into the dermis towards the epidermis indicating a chemoattractant gradient derived from activated skin cells (**Figure 2C**).

### Th1/17 and Tc1/17 cells induce psoriatic-like phenotype in human skin equivalents

**Figure 3:**
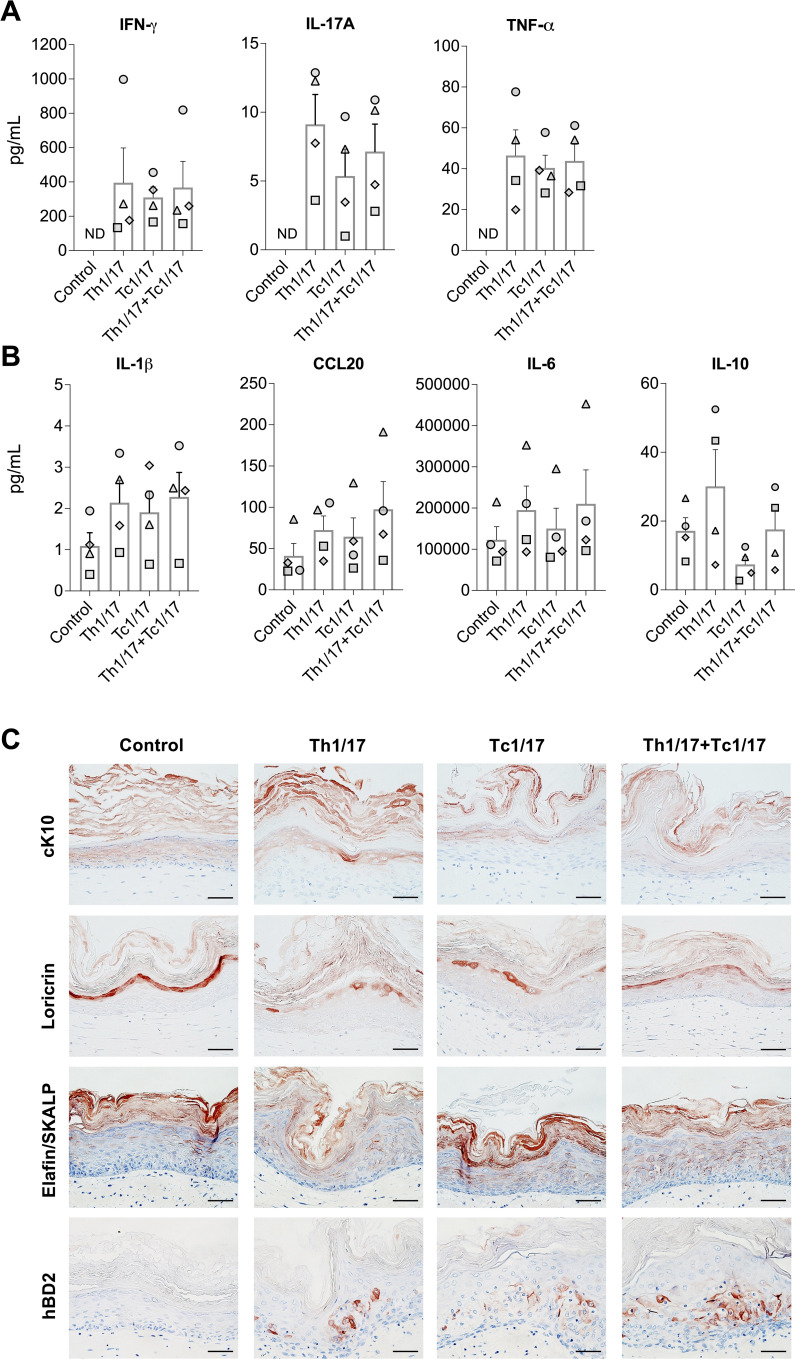
Polarized Th1, Th17, Tc1 and Tc17 cells induce inflammation and histopathological features in human full thickness skin equivalents. Titers of the psoriasis-associated cytokines and chemokine **(A)** IFN-γ, IL-17A, TNF-α and **(B)** IL-1β, CCL20, IL-6 and IL-10 in culture medium of hFTSEs with polarized Th1/17, Tc1/17 or combined Th1/17+Tc1/17 T cells after three days of co-cultures compared to T cell-free hFTSE control cultures. Bar diagrams show the mean ± SEM values of n=4 independent experiments. Each shape represents an independent experiment using different skin- and T cell donors and the mean of intra-experimental triplicates. **(C)** IHC staining of epidermal differentiation and structural markers (cK10 and loricrin, respectively) and antimicrobial peptides (Elafin/SKALP and hBD2) in epidermal keratinocytes of hFTSEs with T cells compared to T cell-free control cultures. Pictures are representatives of n≥2 independent experiments. Scale bar = 50 µm.

Psoriasis is driven by skin:T cell interplay mediated by soluble factors. The release of psoriasis-related cytokines was evaluated in the culture supernatant of hFTSEs incorporated with three combinations of T cell subsets (Th1/17, Tc1/17, or Th1/17+Tc1/17) compared to T cell-free hFTSEs control cultures (**Figures 3A, B**). After three days of co-culture, the expression of IFN-γ, IL-17A, and TNF-α was observed only in hFTSEs integrated with T cells but was not detected in the T cell-free control cultures in all donors analyzed (**Figure 3A**). Moreover, the levels of IL-1β, CCL20 and IL-6 were elevated in all T cell combinations compared to T cell-free control in all donors (**Figure 3B**). Due to a high inter-donor variability and a limited sample size, descriptive statistics was applied to describe donor specific response to the different T cell cultures conditions. All donors consistently showed elevated cytokine levels upon T cell integration across all conditions. In two of four donors, levels of the anti-inflammatory cytokine IL-10 increased in the presence of Th1/17 cells and decreased in all donors in the presence of Tc1/17 cells compared to T cell-free control. Of note, mechanical stress from the insert passage caused an increase of IL-1β, CCL20, IL-6 and IL-10 secretion by fibroblasts and keratinocytes in the T cell-free hFTSE control cultures compared to hFTSE non-transfer control cultures that did not undergo insert passage (**Supplementary Figure 1**). Histopathological analysis of hFTSE was employed to assess the influence of psoriasis associated T cells on epidermal keratinocytes differentiation and barrier integrity (**Figure 3C**). In healthy human skin, differentiated keratinocytes in the suprabasal layers of the epidermis but not basal keratinocytes express cytokeratin 10 (cK10). cK10 distribution in hFTSEs tissue sections resemble healthy skin in conditions without T cells. All T cell subset combinations resulted in decreased expression of cK10 in the epidermis with cK10 being located only in the uppermost epidermal layers with thickened cK10 negative layers below. Loricrin, a skin structural protein expressed in late differentiating keratinocytes of the granular layer, was decreased in all T cell-populated hFTSEs with it becoming non-homogenously expressed along the upper granular layer. The expression of psoriasis related AMPs such as Elafin/SKALP and human β-defensin 2 (hBD2) were upregulated in the epidermal keratinocytes of hFTSE integrated with T cells. Staining of Elafin/SKALP was limited to a weak sporadic expression in the upper suprabasal layers of the epidermis in the T cell-free control cultures but extended to the lower suprabasal layers in T cell populated hFTSEs. hBD2 was detected in the suprabasal layers of the T cell populated hFTSEs, but absent or insignificantly expressed in the T cell-free control cultures.

### Skin equivalents with Th1/17 and Tc1/17 cells were differentially affected by apremilast treatment

**Figure 4:**
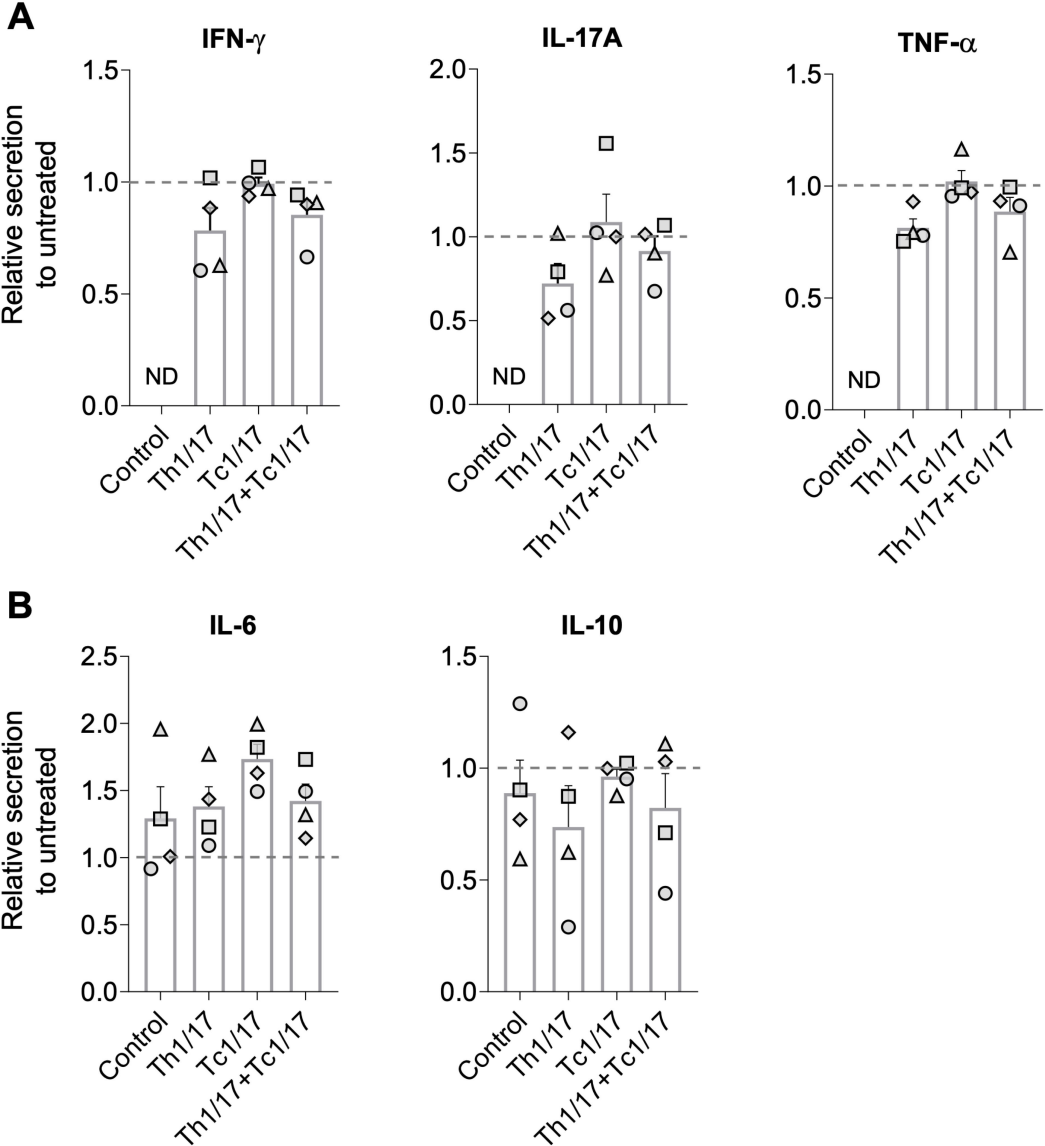
PDE4 inhibitor alters inflammatory cytokine levels in T cell-incorporated human full thickness skin equivalents. 2.5 µM PDE4 inhibitor (apremilast) was administered to the culture medium of hFTSEs on the first day of T cell integration. Concentrations of psoriasis-related cytokines **(A)** IFN-γ, IL-17A, TNF-α and **(B)** IL-6 and IL-10 in the supernatant of the hFTSEs were analyzed after three days of hFTSE:T cell co-culture. Bar diagrams show the mean ± SEM values of cytokine secretion in treated T cell-bearing hFTSE cultures and non-T cell bearing hFTSE control cultures relative to untreated cultures of the same condition. Each shape represents an independent experiment (n=4) using different skin- and T cell donors and the mean of intra-experimental triplicate.

To assess the drug efficacy on psoriasis-like inflammation in our hFTSE models, we applied apremilast, an immunosuppressive phosphodiesterase 4 (PDE4) inhibitor used for the treatment of psoriasis, to the hFTSE culture medium during T cell integration. The degree of inflammation in hFTSEs with and without polarized T cells was assessed by quantifying cytokine secretion in drug-treated cultures relative to untreated cultures of the same condition, after three days of co-culture (**Figure 4**). Apremilast administration reduced the secretion of IFN-γ, IL-17A and TNF-α by Th1/17 cells in at least three out of four donors but not by Tc1/17 cells (**Figure 4A**). IL-6 elevation was observed in all T cell populated hFTSE constructs from all donors and in two out of four donors of the T cell-free control hFTSE upon apremilast application. Notably, one donor of keratinocytes and fibroblasts (grey triangle) displayed high IL-6 expression at baseline (untreated control culture) and responded with strong IL-6 induction to T cell integration and drug application (**Figure 4B**). Apremilast also did not influence secretion of IL-10 by Tc1/17 and only Th1/17 donors with comparatively high levels of these cytokines displayed drug response (**Figure 4B**, **Supplementary Figure S2**). Ultimately, the cytokine profile across the different T cell cultures revealed that cytokine production in Th1/17 cultures was predominantly responsive to drug treatment among donors, whereas Tc1/17 cultures tended to remain unresponsive.

## Discussion

Current experimental models of psoriasis are predominantly based on murine systems, including topical imiquimod application, intradermal IL-23 injection, keratinocyte-specific IL-17 expression, or overexpression of components of the IL-23/IL-17 signaling axis (27). Murine models provide substantial advantages for mechanistic studies due to their genetic manipulability, systemic immune integration, and suitability for longitudinal *in vivo* analyses. However, their translational value is limited by fundamental interspecies differences in skin architecture, microbiome composition, and immune cell distribution (34). Notably, murine skin exhibits a relative predominance of γδ T cells, whereas human psoriatic skin is primarily infiltrated by αβ T cells (35). To address these limitations, incorporation of defined human immune cell subsets into human skin equivalents is essential for the development of advanced experimental platforms that more accurately recapitulate human skin immunity and the pathomechanisms of inflammatory skin diseases. Previous studies have demonstrated that the integration of Th1 and Th17 CD4^+^ T cells into skin models can mimic psoriasis-like inflammatory processes (29, 30). Given the growing evidence supporting a central role for CD8^+^ T cells in psoriasis pathogenesis (10–13), we here elucidate the development of an immunocompetent hFTSE incorporating CD4^+^ and as well CD8^+^ T cell subpopulations in order to study the psoriasis pathogenesis and effects of anti-psoriatic drug administration.

The interplay between keratinocytes and distinct T cell subpopulations forms a core signaling axis in psoriasis, driving psoriasis-specific self-sustaining inflammation. It has been demonstrated that both distinct CD4^+^ and CD8^+^ T cell subpopulations secrete proinflammatory cytokines such as IFN-γ, IL-17, and TNF-α (6, 14, 15, 17, 19). These cytokines can activate keratinocytes, leading to hyperproliferation and the release of chemoattractants and inflammatory mediators such as CCL20, IL-1β and IL-6 (9, 36, 37). This, in turn, induces a subsequent inflammatory response in immune and tissue cells, as well as the recruitment of inflammatory T cells into the dermis, thereby establishing a chronic inflammatory niche characteristic of psoriasis (38).

*In vitro* polarization of Th1/Tc1 and Th17/Tc17 cells was confirmed by expression profiles for IFN-γ, IL-17A, and IL-17F - mirroring expression seen in *ex vivo* isolated T cells. Interestingly, IL-13, traditionally associated with type 2 responses, was also secreted by Th17 and Tc17 cells, consistent with recent reports showing IL-13 production by type 17 subsets (15). In inflammatory skin, IL-13 promotes CCL26 release from keratinocytes, which recruits CCR4^+^ T cells - including type 17 and type 22 subsets - into lesional skin (15, 39–41). Notably, despite this shared chemotactic axis, type 2 T cells are not prominently enriched in psoriatic lesions, suggesting the presence of additional regulatory mechanisms that selectively shape the local T cell composition. Recent findings have further delineated IL-22-producing T cells as a distinct population from Tc17 cells, and accordingly, we observed minimal IL-22 production in our type 17-polarized cultures (42).

T cell–derived IFN-γ and IL-17 have been shown to induce chemokine expression in epidermal keratinocytes - specifically CXCL9/CXCL10, recruiting further type 1 T cells via CXCR3 - and CCL20, recruiting further type 17 T cells via CCR6 (9, 43). Consistent with this, our data demonstrates T cell migration towards the epidermis in all three conditions (hFTSEs integrated with Th1/17, Tc1/17 or Th1/17 and Tc1/17 combined). In addition, the expression of key inflammatory markers (IFN-γ, IL-17A, TNF-α, IL-1β, CCL20 and IL-6) was upregulated compared to T cell-free control cultures. Importantly, IFN-γ, TNF-α and IL-17A were only detected in T cell-populated hFTSEs, indicating retained T cell functionality post-integration. Additionally, the upregulation of CCL20, IL-1β, and IL-6 indicated active crosstalk between T cells and skin cells. Interestingly, IL-10 was elevated in half of the hFTSEs with Th1/17 cells and reduced in the other half. IL-10 is known to act as a self-regulatory feedback mechanism in IFN-γ–producing Th1 cells (44), suggesting that its expression may originate from activated T cells in response to elevated levels of inflammatory cytokines, such as IFN-γ. Notably, hFTSEs incorporated with CD8^+^ T cells (Tc1/17) exhibited lower levels of inflammatory cytokines in most donors. Although Tc1/17 cultures showed robust cytokine production prior to integration, their activity declined post-integration. Upon antigen stimulation *in vivo*, naïve CD8^+^ T cells more rapidly become activated, proliferate and differentiate into effector cells than CD4^+^ T cells (45). Therefore, their cytokine production may also decline earlier than that of CD4^+^ T cells in the absence of continued stimulation.

T cells integration into hFTSEs induced key histopathological features of psoriatic lesions including impaired epidermal differentiation and the upregulation of AMPs. Reduced expression of cK10 was observed in the lower suprabasal layers of a lesional psoriatic epidermis, as well as in keratinocyte cultures applied with psoriasis-associated cytokines IFN-γ, IL-17A and IL-22 (46, 47). Accordingly, we observed reduced expression of cK10 in the lower suprabasal layers of T cell-populated hFTSE. Similarly, loricrin, a key epidermal barrier protein downregulated by TNF-α, was reduced in cultures with T cell-driven TNF-α expression (48). Excessive expression of AMPs, such as hBD2 and Elafin/SKALP in epidermal keratinocytes is characteristic to psoriatic lesions (49). Previous *in vitro* studies showed that hBD2 and Elafin/SKALP upregulation in epidermal keratinocytes is induced by inflammatory cytokines IFN-γ, IL-17, TNF-α, IL-1β and IL-6 and TNF-α, respectively (50, 51). In line with these findings, AMPs such as hBD2 and Elafin/SKALP - both regulated by inflammatory cytokines - were markedly upregulated in the epidermis of inflamed hFTSE.

To investigate the potential use of our immunocompetent hFTSEs as a drug testing platform, we administered apremilast, a common psoriasis drug, shown to reduce psoriasis-associated proinflammatory cytokine in a T cell dependent manner (22, 52). While cytokine suppression (IFN-γ, TNF-α, IL-17A) did not reach statistical significance due to inter-donor variability, consistent downward trends were observed in most CD4^+^ T cell–containing cultures. Interestingly, CD8^+^ T cells were unresponsive, potentially due to lower PDE4 subtype expression, limiting drug efficacy (53). This highlights the importance to integrate CD8^+^ T subpopulations in human psoriasis models to improve their translational potential. IL-6 was consistently upregulated post-treatment, aligning with prior studies showing apremilast-induced IL-6 and IL-10 elevation in PBMCs (52, 54). Conversely, IL-10 expression declined in several donor cultures, possibly reflecting reduced upstream inflammatory cytokines and the consequent drop in inflammation-induced autocrine IL-10 signaling (44). The technical complexity of the here used model limits the capacity for conducting a high throughput screening for now, which would have facilitated the analysis of high inter-donor variability. The intra-donor replicates consistency supported the reproducibility of donor-specific drug-response, thus implicating the potential use of this model as a personalized prediction tool for drug testing. The observed inter-donor variability, while limiting statistical power, may reflect the well-known clinical and immunological heterogeneity of psoriasis patients. Such variability is inherent to primary human cell models and may enhance the translational relevance of our findings by capturing patient-specific differences that are not represented in more homogeneous systems (55). Future work should include larger cohorts in order to increase statistical power.

In conclusion, we report for the first time a full-thickness immunocompetent human skin model integrated with *in vitro* polarized human CD4^+^ and CD8^+^ T cell subpopulations exhibiting inflammatory features characteristic of psoriasis. Using this model as a substance testing platform, we were able to demonstrate T cell subset-dependent differences in the effect of the psoriasis drug apremilast. This model shows promise as a disease modelling tool for investigating CD4^+^ and CD8^+^ T cell immunoreactivity in psoriatic skin as well as a testing platform for assessing the efficacy and safety of drugs in preclinical settings. While introducing CD8^+^ T cell subsets is a critical step towards a more physiological psoriasis model, psoriatic inflammation is influenced by numerous immune cells, including DCs, monocytes, mast cells, neutrophils, B cells, regulatory- and γδ-T cells (38). In particular, IL-22 producing T cell subsets, which are also highly enriched in psoriatic skin and have been strongly correlated with the recurrence of psoriatic inflammation, should be included (16). Introducing further skin components such as appendages, subcutaneous layer and a perfused vascular and neuronal network is an important future aspect to support more accurate recapitulation of *in vivo* pathological mechanism and drug metabolism (56). In addition, incorporation of a functional vascular compartment would enable modeling of endothelial activation, leukocyte extravasation, angiogenesis, and cytokine-mediated vascular inflammation, thereby addressing key aspects of psoriasis-associated vascular remodeling and cardiovascular comorbidity. Finally, the integration into microfluidic multi-organ-chip systems could enable the investigation of complex physiological or pathological systemic processes, as well as a more physiological immune cell migration and infiltration into an inflamed tissue model.

## Data Availability

The datasets presented in this study can be found in online repositories. The names of the repository/repositories and accession number(s) can be found below: https://doi.org/10.5281/zenodo.15641259.

## Supplementary material

Supplementary Figure 1 Baseline cytokine release in human full thickness skin equivalents undergoing insert replacement. Control cultures of hFTSE were transferred onto a T cell-free insert (T cell-free control) while non-transfer control cultures were maintained in their original inserts. The impact of insert-replacement procedure on hFTSEs inflammatory profile was evaluated by comparing the level of inflammation between non-transfer control and T cell-free control cultures. Concentrations of inflammatory mediators (IFN-γ, IL-17A, TNF-α, IL-1β, CCL20, IL-6 and IL-10) in hFTSE supernatant of both control cultures were analyzed on day three post-insert replacement. Bar diagrams show the mean ± SEM values of n=4 independent experiments. Each shape represents an independent experiment using different skin cells donors (matching keratinocyte and fibroblasts) and the mean of intra-experimental triplicates.

Supplementary Figure 2 Intra-experimental reproducibility of PDE4 inhibitor-induced alteration of inflammatory cytokine levels in T cell-incorporated human full thickness skin equivalents. Four independent experiments were carried out to evaluate PDE4 inhibitor-response in hFTSEs, integrated with psoriasis-associated T cells. Concentrations of psoriasis-related cytokines IFN-γ, IL-17A, TNF-α, IL-6 and IL-10 in the supernatant of the hFTSEs were analyzed after three days of hFTSE:T cell co-culture. Each diagram represents one independent experiment using different skin- and T cell donors. Bar diagrams show the mean ± SEM values of ≤3 intra-experimental replicates.
