# Supplementary figures and images for: CD4+ and CD8+ T cells contribute to psoriasis phenotype when integrated into human skin equivalents for drug testing
Source: Front Immunol. 2026 Apr 10;17:1790531. doi: 10.3389/fimmu.2026.1790531 (PMC13106556; doi:10.3389/fimmu.2026.1790531)

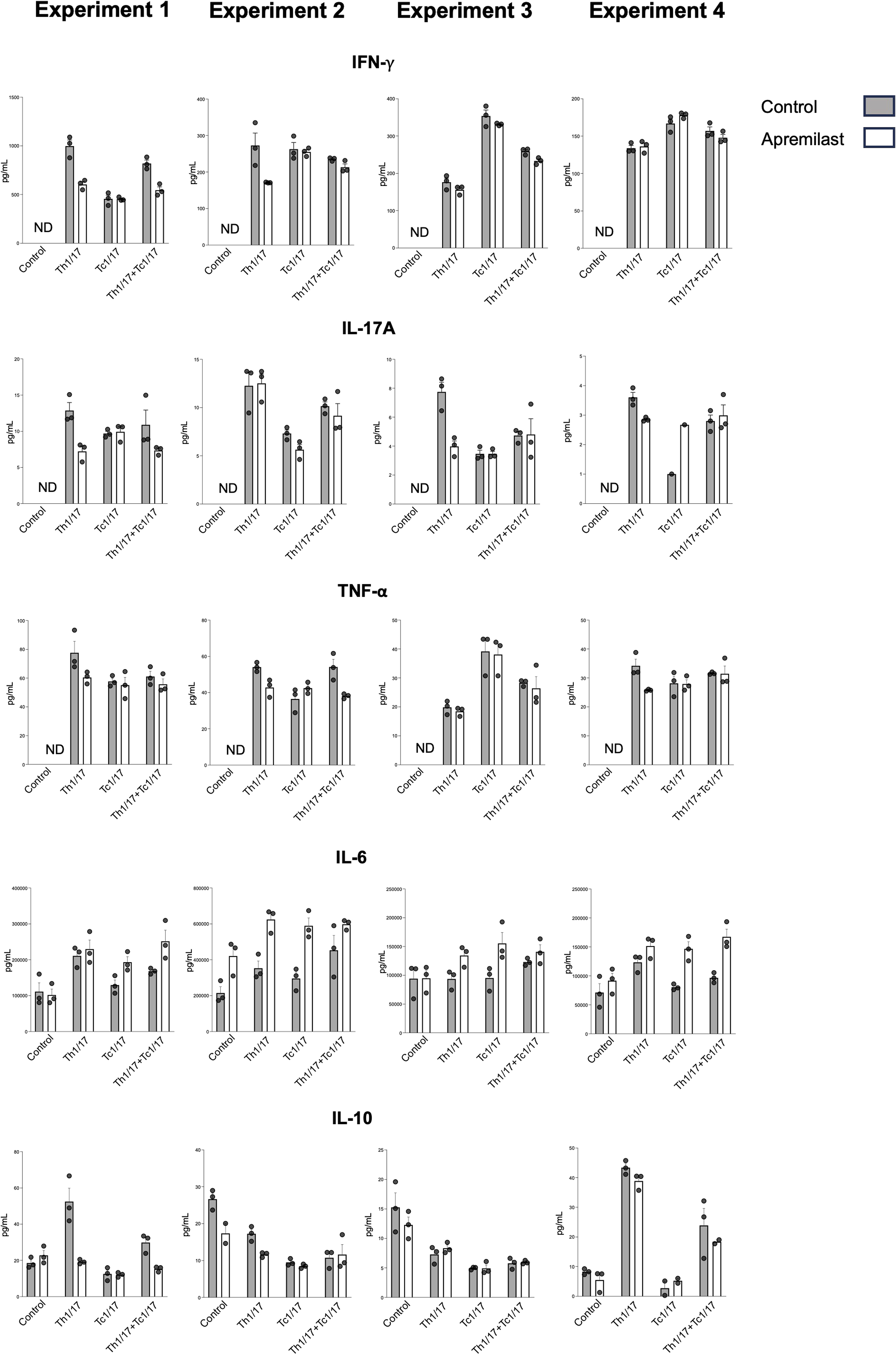

Supplement: Supplementary Figure 2 — Intra-experimental reproducibility of PDE4 inhibitor-induced alteration of inflammatory cytokine levels in T cell-incorporated human full thickness skin equivalents. Four independent experiments were carried out to evaluate PDE4 inhibitor-response in hFTSEs, integrated with psoriasis-associated T cells. Concentrations of psoriasis-related cytokines IFN-γ, IL-17A, TNF-α, IL-6 and IL-10 in the supernatant of the hFTSEs were analyzed after three days of hFTSE:T cell co-culture. Each diagram represents one independent experiment using different skin- and T cell donors. Bar diagrams show the mean ± SEM values of ≤3 intra-experimental replicates. [file Image2.jpeg]
